# Characterizing Variation of Branch Angle and Genome-Wide Association Mapping in Rapeseed (*Brassica napus* L.)

**DOI:** 10.3389/fpls.2016.00021

**Published:** 2016-02-04

**Authors:** Jia Liu, Wenxiang Wang, Desheng Mei, Hui Wang, Li Fu, Daoming Liu, Yunchang Li, Qiong Hu

**Affiliations:** ^1^Key Laboratory of Biology and Genetic Improvement of Oil Crops, Ministry of Agriculture, Oil Crops Research Institute of the Chinese Academy of Agricultural SciencesWuhan, China; ^2^Agricultural Sciences Institute of Lu'an MunicipalLu'an, China

**Keywords:** *Brassica napus* L., branch angle, genetic variation, association mapping, multiple environments, candidate genes

## Abstract

Changes in the rapeseed branch angle alter plant architecture, allowing more efficient light capture as planting density increases. In this study, a natural population of rapeseed was grown in three environments and evaluated for branch angle trait to characterize their phenotypic patterns and genotype with a 60K *Brassica* Infinium SNP array. Significant phenotypic variation was observed from 20 to 70°. As a result, 25 significant quantitative trait loci (QTL) associated with branch angle were identified on chromosomes A2, A3, A7, C3, C5, and C7 by the MLM model in TASSEL 4.0. Orthologs of the functional candidate genes involved in branch angle were identified. Among the key QTL, the peak SNPs were close to the key orthologous genes *BnaA.Lazy1* and *BnaC.Lazy1* on A3 and C3 homologous genome blocks. With the exception of Lazy (*LA*) orthologous genes, *SQUMOSA PROMOTER BINDING PROTEIN LIKE 14* (*SPL14*) and an auxin-responsive *GRETCHEN HAGEN 3* (*GH3*) genes from *Arabidopsis thaliana* were identified close to two clusters of SNPs on the A7 and C7 chromosomes. These findings on multiple novel loci and candidate genes of branch angle will be useful for further understanding and genetic improvement of plant architecture in rapeseed.

## Introduction

Rapeseed (*Brassica napus* L. 2n = 4 × = 38, AACC genomes) is a widely cultivated oil crop throughout the world. Yield improvement and mechanized harvesting are extremely urgent recently for the demands of rapeseed producers in addition to the edible oil and biofuel industries (Diepenbrock, 2000). The ideotype of a plant is defined as the spatial distribution of various architectures which is an important agronomic character that affects photosynthesis and seed yields (Donald, 1968; Mansfield and Mumm, 2014). The ideotype can influence photosynthesis, plant growth, and seed yield due to the least competition among the individuals in a population (Wang and Li, 2005). The ideotype is determined by a combination of architecture factors including branch angle (BA), plant height (PH), first branch height (FBH), inflorescence length (IL), and branch number (BN; Mei et al., 2009; Shi et al., 2009; Xu et al., 2014). In particular, branch angle, or the angle between branch and erect primary stem, has long attracted the attention of breeders because of the significant contribution of this trait to plant architecture.

High yields can be achieved through high plant density with a small branch angle, which determines the plant's ability to grow and capture light efficiently. Wang and Li (2008) reported in rice more upright and dense leaves not only improve light capture but also improve the accumulation of leaf nitrogen for grain filling. The branch (or leave) angle has also been studied in maize, cotton, and other important crops to achieve an ideal plant architecture to improve yields (Li et al., 2007; Jin et al., 2008; Song and Zhang, 2009; Ku et al., 2011; Tian et al., 2011; Bai et al., 2012). Several quantitative trait loci (QTL) related to leaf angle have been identified genetically in maize.

In recent years, genome-wide association (GWA) mapping has become a powerful tool for identifying important genes associated with complex traits, which has been used with success in model and non-model plants (Breseghello and Sorrells, 2006; Atwell et al., 2010; Huang et al., 2010; Zhao et al., 2011). Additionally, with the dramatically decreasing cost of genome sequencing and rapid developments in genome analysis, *Brassica* A genome sequence from *Brassica rapa* and *Brassica* C genome sequence from *Brassica oleracea* have been published (Wang et al., 2011; Liu et al., 2014). It is important to complete the rapeseed (*B. napus*, AC genome) genome sequencing (Chalhoub et al., 2014). Single nucleotide polymorphisms (SNPs) are abundant and evenly distributed throughout the genomes, which is more conducive to GWA. In 2012, a *B. napus* 60K SNP Infinium genotyping array was produced and applied by the international *Brassica* SNP consortium in cooperation with Illumina Inc. San Diego, CA, USA (Snowdon and Iniguez Luy, 2012; Edwards et al., 2013). Studies about GWAS in rapeseed have gained attention in recent years and various traits including flowering time, seed weight and seed quality have been dissected (Harper et al., 2012; Cai et al., 2014; Li et al., 2014; Lu et al., 2014; Raman et al., 2014; Wang et al., 2014). However, no report was found on association mapping for rapeseed branch angle to our knowledge.

Numerous genes are known to influence the branch (tiller) angle in model and other crops. For instance, *OsTAC1* was reported to play a critical role in controlling rice architecture (Yu et al., 2007). Li et al. (2007) reported that *LAZY1* regulates shoot gravitropism through which the rice tiller angle is controlled. Enlarged branch angles with agravitropic shoots were similarly also found in *Atlazy1* mutant of *Arabidopsis* (Yoshihara et al., 2013). Dong et al. (2013) identified maize *ZmLA1* gene as a functional ortholog of *LAZY1* in rice and *Arabidopsis*. The regulation of branch angle is a combination of environmental factors and hormone homeostasis (Lomax, 1997). Auxin may be the primary hormone involved in shoot gravitropism (Robert and Friml, 2009), which is a key process in determining branch (leave) angle. The member of *GH3* family plays crucial roles in auxin homeostasis in relation to leaf inclination control (Zhao et al., 2012). Recent evidence showed that new strigolactone plant hormones regulated rice tiller angle by attenuating shoot gravitropism through the inhibition of auxin biosynthesis (Sang et al., 2014). Although interaction of auxin and strigolactone plays an important role in shoot gravitropism, the key genes by which the two hormones regulate shoot gravitropism is not yet identified. However, knowledge of the genes that control genetic variation for branch angle of rapeseed is limited.

In this study, a panel of 143 elite rapeseed accessions was analyzed by the 60 K *Brassica* Infinium SNP array. The branch angle was measured in 2013–2014 at three environments. The SNPs in the array were *in silico* mapped to A and C genomes of Darmor-*bzh B. napus* genome “pseudomolecules” to obtain their hypothetical position. The aims were (1) to gain the population structure and genetic diversity in elite germplasms; (2) to detect QTL controlling branch angle and mine for elite alleles; and (3) to predict the candidate genes.

## Methods

### Plant material and field experiments

A total of 143 rapeseed accessions were used for an association analysis in this study. According to the information from field observations, the accessions were classified to three different germplasm types, i.e., spring oilseed rape (OSR) (13), semi-winter OSR (124), and winter OSR (6). Based on their origins, 112 accessions originated from China, 24 from Oceania, 5 from Europe, 1 from North America, and 1 from India (Supplementary Table 1). The seeds from all the accessions were collected, stored and supplied by Oil Crops Research Institute of Chinese Academy of Agricultural Sciences (OCRI-CAAS). In recent decades, these accessions have been widely used as parents in breeding programs.

The experiments were conducted at Yangluo Agronomic Experimental Station of OCRI-CAAS (28°42′N, 112°33′E), Wuhan, China, during the 2013 and 2014 winter growing season and at Lu'an Experimental Station (31°73′N, 116°52′E) in Anhui, China, during the 2013 winter growing season. In each environment, the experiment was conducted in randomized complete blocks with two replicates. Each plot contained three rows with 54 individuals, setting as 33 cm between rows and 11 cm between plants within each row, with a planting density of 270,000 plants/ha. All experiments were performed under local field management and cultivation conditions.

### Trait measurements and statistical analysis

In each plot, five typical plants were harvested for branch angle measurement at the mature stage. The branch angle (BA) was measured as the angle between the main stem and the branch. BA was scanned with a digital camera (SONY DSLR-A350; SONY, Japan). The angle value from the images was obtained using AutoCAD software (Autodesk Inc., San Rafael, CA). The average BA value of five individual plants for each plot was calculated as the final phenotypic value. In addition, the best linear unbiased estimators (BLUPs) across all three environments were predicted by assuming fixed genotypic effects to minimize the effects of environmental variation. Finally, each environment and BLUP value was used as a phenotype for the association analysis.

Statistical analysis of the data was performed by using PROC MEANS in SAS software, Version 9.3 (2000, SAS Institute Inc., Cary, NC, US). Analysis of variance (ANOVA) was conducted by using PROC GLM to determine the effects of block, environment, genotype and genotype-environment interactions. Correlation coefficients were obtained by using PROC CORR. The Broad-sense heritability (*H*^2^) was calculated as *H*^2^ = σg^2^/(σg^2^ + σgl^2^/*n* +σe^2^/*nr*), in which σg^2^, σgl^2^, σe^2^, *r*, and *n* represent the estimated variances for the genetic effects, genotype-environment interactions, random errors, number of replications and number of environments, respectively. The estimated variances for σg^2^, σgl^2^, and σe^2^ were obtained by ANOVA.

### SNP genotyping, quality control and *In silico* mapping of SNPs

The genotype of 143 accessions was detected with a *Brassica* 60K Illumina Infinium SNP array according to the work flow by Emei Tongde Co. All of the SNP data were clustered and called up automatically by using Illumina BeadStudio genotyping software. The SNP quality was checked and comparable with previous studies (Li et al., 2014; Wang et al., 2014). The low quality SNP loci (call rate < 80% and/or minor allele frequency < 0.05) in all accessions were deleted from the results. Out of 52,157 SNPs in the array, 2836 that had a zero call frequency of AA or BB were excluded according to the quality control. Using a cut-off for missing data >0.2 and MAF < 0.05, 1860 and 1909 SNPs were filtered, respectively, reducing the number of SNPs to 38,063.

SNP mapping was performed as previously reported (Altschul et al., 1990). In brief, BLAST search against the “pseudomolecules” representative of the *B. napus* genome (version 4). Only the top BLAST hits with an *E*-value cut-off of 1*E*-15 against the pseudomolecules were retained, while BLAST matches to multiple loci with the same *E*-value were deleted. A final set of 34,469 high-performing SNPs was used for all the analyses.

### Population genetic analysis

Nei's genetic distance matrix for all SNPs is the distance and it was calculated to build unrooted neighbor-joining trees by using PowerMarker (Liu and Muse, 2005). The result was visualized by using FigTree software based on 34,469 SNPs (http://tree.bio.ed.ac.uk/software/figtree/). Kinship (K) matrix used to compare all the pairs of the 143 accessions was calculated with 2434 informative SNPs with a MAF > 0.2 by using the SPAGeDi software package (Hardy and Vekemans, 2002). All negative kinship values that were found between two individuals, which indicates that there was less of a relation than expected between two random individuals, were transformed to zero (Yu et al., 2005).

A total of 2434 SNPs [minor allele frequency (MAF) ≥ 0.2] that were evenly distributed across the whole genome were selected to create the population structure inferred by using the STRUCTURE v2.3.4 software package (Pritchard et al., 2000). Iterations were performed 100,000 times by using a burn-in length of 100,000 MCMC (Markov chain Monte Carlo) with the admixture and related frequency model. Five independent runs were performed with *K*-values (the putative number of populations) ranging from 1 to 10. The optimal *K*-value was determined by taking the log probability of the data [LnP(D)] and an *ad hoc* statistical Δk based on the rate of change for LnP(D) between successive *k*-values as described by Evanno et al. (2005). The cluster membership coefficient matrices of replicate runs from STRUCTURE were integrated to obtain a Q matrix by using CLUMPP software (Jakobsson and Rosenberg, 2007) and graphically displayed by using the DISTRUCT software package (Rosenberg, 2004). Accessions with a probability of membership >0.7 were assigned to corresponding clusters, and those < 0.7 were assigned to a mixed group. The population structure matrix (Q) was generated for further analyses. Linkage disequilibrium (LD) parameter (*r*^2^) for estimating the degree of LD between pair-wise SNPs (MAF ≥ 0.05) was calculated using the software TASSEL 4.0 (Bradbury et al., 2007).

### Association analysis

Two different models were used to test associations. The first model was a simple and general linear model (GLM) without controlling for Q and K, containing only the SNP that was tested as a fixed effect. The second model was a mixed linear model (MLM) where, in addition to testing the SNP, the population structure (Q) and relative kinship matrix were included as fixed and random effects, respectively. Analyses were performed by using TASSEL 4.0 software, for which the optimum compression and population parameters previously determined (P3D) variance component estimation were implemented to decrease the computing time for the large data set (Zhang et al., 2010).

The significance of associations between SNPs and trait was based on a threshold of *p* < 2.90 × 10^−5^ [i.e., -log_10_(*p*) = 4.5]. The threshold is 2.90 × 10^−5^ at a significant level of 1% after Bonferroni multiple test correction (1/34,496). Furthermore, we applied the false discovery rate (FDR) technique. We calculated an FDR *q*-value for each association test by using the software QVALUE (Dabney and Storey, 2004). The FDR *q*-value of the significant SNP with the lowest test statistic (*P* < 0.05) provided an estimate of the proportion of false positives among the significant associations. The significant value and the marker effect for each SNP were exported, and a Manhattan plot was generated in the R package qqman (Turner, 2014, http://cran.r-project.org/web/packages/qqman/).

Stepwise regression was performed to detect the effect of multiple alleles with different functional polymorphisms on branch angle and to estimate the total variance explained (*R*^2^) by using the *lm* function in R (Ihaka and Gentleman, 1996).

## Results

### Phenotypic variation of branch angle

**Figure 1:**
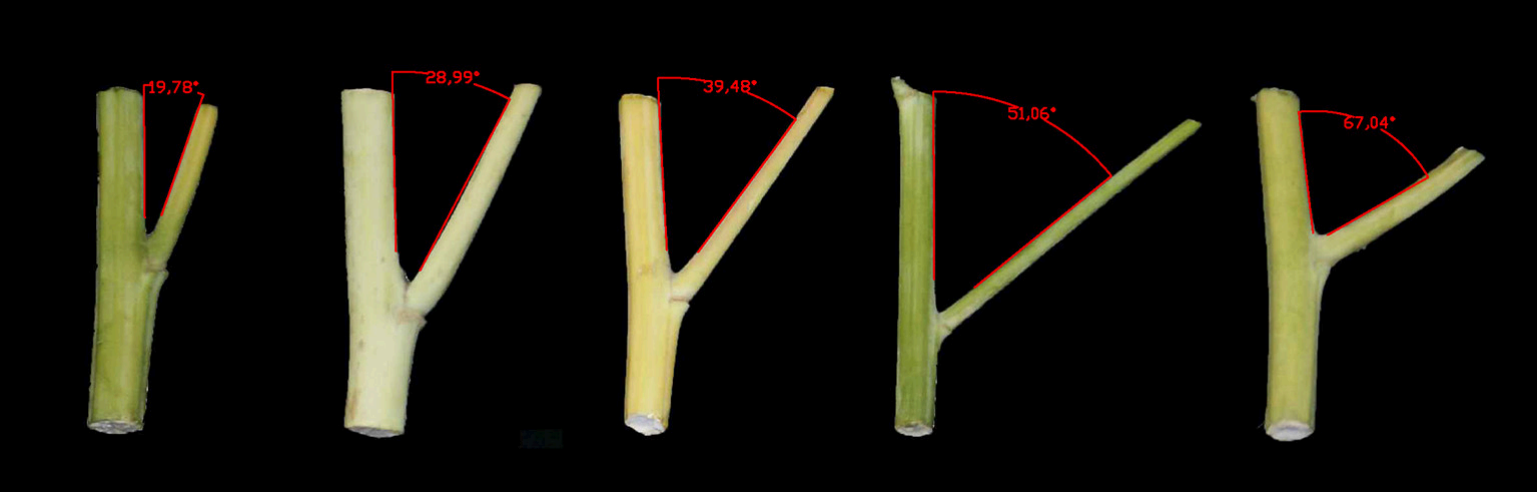
**Branch angle was measured using AutoCAD software**.

**Figure 2:**
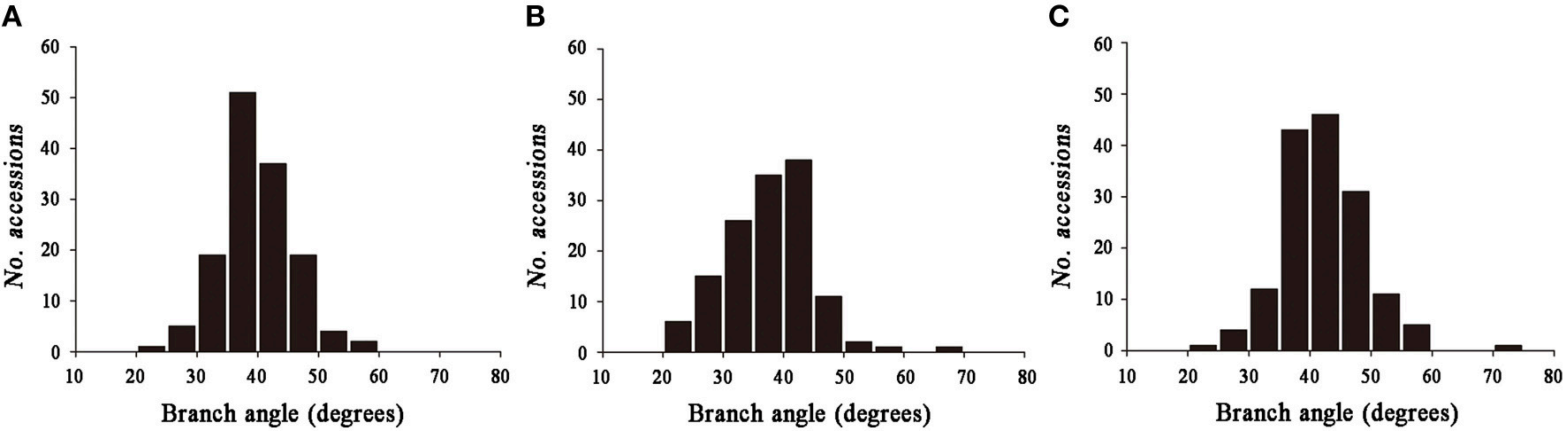
**The frequency distribution for branch angle in three environments. (A) 2013YL; (B) 2014YL; (C) 2013LA**.

Significant variation was observed among the 143 rapeseed accessions in the three environments studied, with branch angles ranging from 20 to 70° (Figure 1, Table 1). In three environments Yangluo-2013, Yangluo-2014, and Lu'an-2013, the natural population exhibited average branch angle values (±SD) of 39.93 ± 5.98, 37.52 ± 7.26, and 42.30 ± 6.65, respectively. The frequency distributions of branch angle in the natural population are summarized in Figure 2. Branch angle ranged from 22.32 to 59.57, 20.04 to 69.09, and 22.98 to 70.77 in three environments, respectively.

**Table 1:** **Phenotypic characteristics for branch angle in 143 rapeseed accessions**.

**Environment**	**Min**	**Max**	**Mean ± SD**	**CV%**	**Kurtosis**	**Skewness**	**Correlation coefficient**		
							**13YL**	**14YL**	**13LA**
YL-2013	22.32	59.57	39.93 ± 5.99	14.99	0.91	0.08		0.65^**^	0.68^**^
YL-2014	20.04	69.09	37.52 ± 7.26	19.34	1.92	0.31			0.56^**^
LA-2013	22.98	70.77	42.30 ± 6.65	15.71	2.22	0.45			

*SD, standard deviation; CV, coefficient of variation; Kurtosis is the distribution of observed data around the mean; Skewness is a measure of the asymmetry of the probability distribution of a real-valued random variable about its mean*.

** *Significant at P < 0.01*.

*YL, Yangluo; LA, Lu'an*.

The two-way ANOVA showed that differences among the lines in the branch angle were highly significant. This finding confirmed that a large number of genetic variation existed in the population. The effects from years and location were significant (Table 2). The broad-sense heritability (*H*^2^) for branch angle was calculated as 93.90%, demonstrating that branch angle in these rapeseed lines was conditioned primarily by genetic factors. The correlation coefficients for branch angle between environments were all relatively high (*r* ≥ 0.557, *P* < 0.01).This observation indicated that branch angle in the 143 lines was relatively consistent across environments.

**Table 2:** **ANOVA and broad-sense heritability for branch angle**.

**Variance resource**	**DF**	**SS**	**MS**	***F*-value**	***H*^2^(%)**
Environments	2	1210.86	605.43	139.56^**^	93.90
Gene	142	18487.16	133.96	30.88^**^	
Rep	1	20.01	20.01	4.61^*^	
G × E	305	6513.11	23.95	5.52^**^	

DF, degree freedom; SS; stdev square; MS, mean square; F-value, F-test value; H^2^, heritability; G × E, genotype by environment interactions;

*, ** *Significant at P < 0.05 and 0.01 probability levels, respectively*.

### SNP genetic diversity and linkage disequilibrium

Supplementary Table 2 gives the information on all of the SNPs. Estimates of an average nucleotide diversity (polymorphism information content or PIC) of 0.366 showed that the overall genetic variation in the accessions studied here represents ~62.9% of the rapeseed diversity. There had the most markers (2745) with a marker density of one per 26.4 kb in C3 linkage group and the fewest markers (932) with a marker density of one per 47.6 kb in C5.

**Figure 3:**
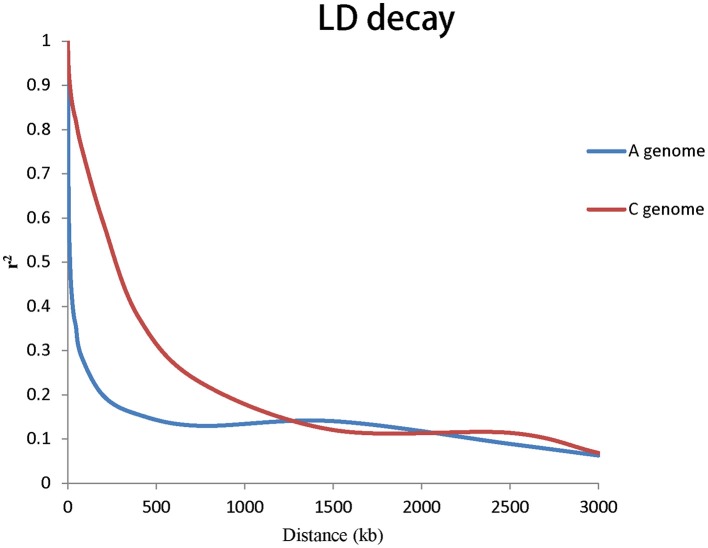
**Genome-wide LD decay of A and C genomes for all the 143 accessions**.

To evaluate the extent of LD, *r*^2^ was used to calculate LD. The genome-wide LD decay of A and C genome for rapeseed germplasms are shown in Figure 3. Taken together, the LD of A genome decayed significantly faster than that of the C genome. We estimated the LD decay, *r*^2^ decayed to 0.2 when the average distance for A genome was 206 kb and *r*^2^ decayed to 0.2 when the average distance for C genome was 949 kb.

### Population structure and relative kinship

**Figure 4:**
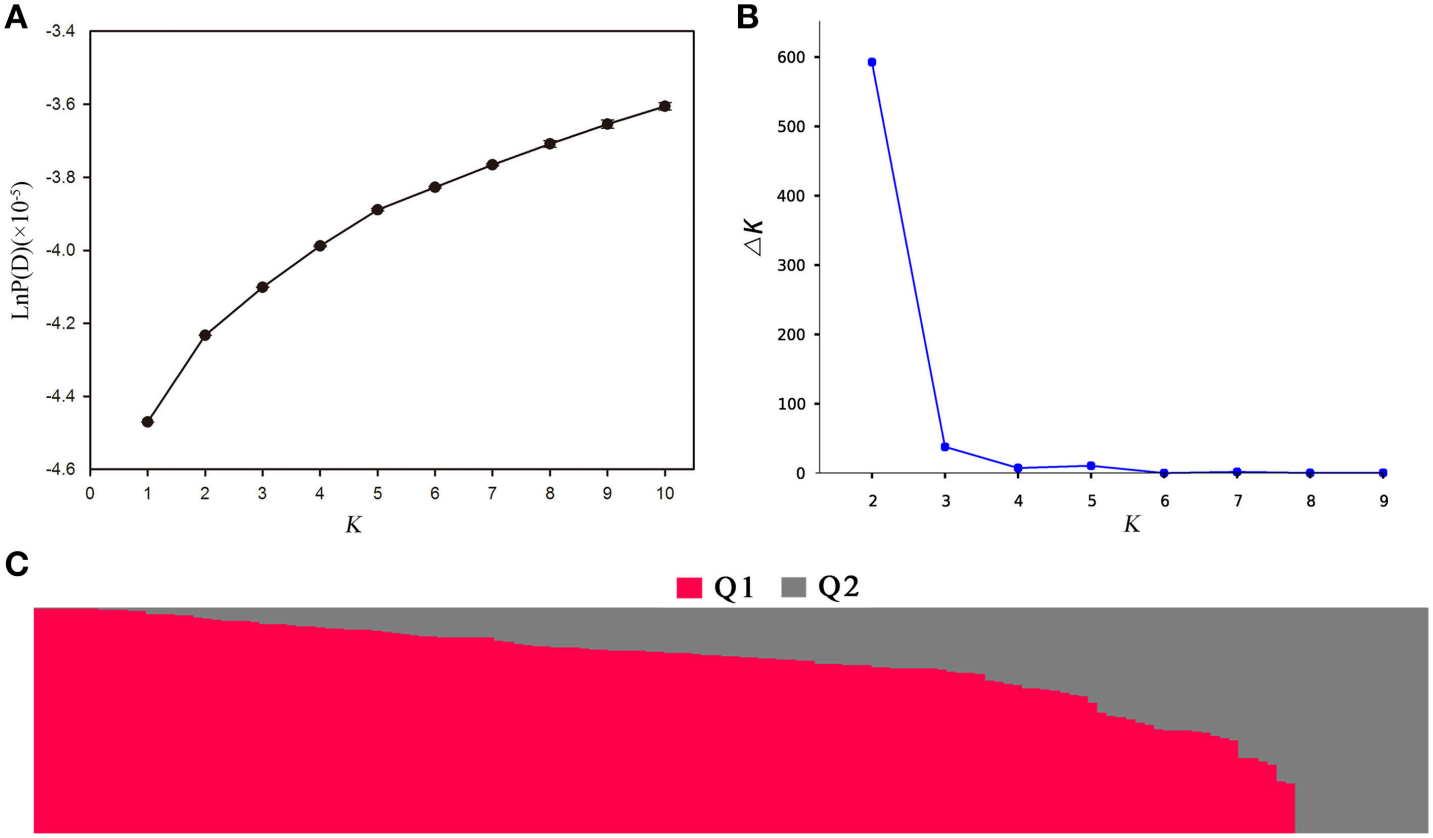
**Population structure analysis of 143 rapeseed accessions by STRUCTURE software. (A)** The estimated LnP(D) of possible clusters (k) from 1 to 10; **(B)** Δk based on the change of LnP(D) between consecutive k; and **(C)** Q1 and Q2 are the composition values belonging to the two sub-populations (*K* = 2) for a given accession which is represented by a vertical bar.

Population structure of the association panel was calculated using 2434 SNPs, and a clustering inference performed with possible clusters (k) from 1 to 10 showed that the most significant change in likelihood occurred when K increased from 2 to 3, and the highest Δ*k*-value was observed at *k* = 2 (Figure 4). Based on the Δk method (Figure 4B), the 143 accessions could be divided into two sub-populations (Figure 4C). By using a probability-of-membership threshold of 70%, 99 and 17 lines were assigned to the two groups, respectively. The remaining 27 lines were classified into a mixed group (Supplementary Table 1). In comparison with a previous study, this population structure classification yields the same results (Harper et al., 2012; Lu et al., 2014; Wang et al., 2014). In addition, the NJ phylogenetic tree based on Nei's genetic distances displayed two clear clades (Supplementary Figure 1), corresponding to the two groups estimated by STRUCTURE. The lines belong to mixed groups were distributed across the whole tree. Tree-based analyses yielded results very similar to those of the STRUCTURE analysis.

**Figure 5:**
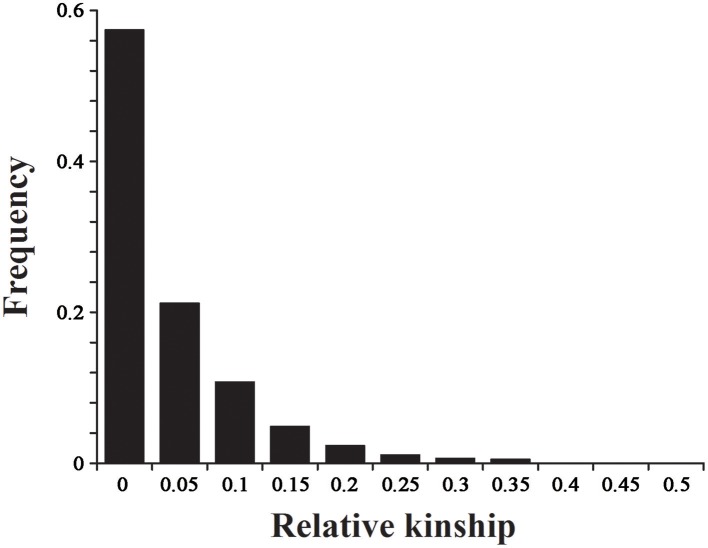
**The distribution for pairwise relative kinship**. Only the kinship values ranging from 0 to 0.5 are shown.

The 2434 informative SNPs with a MAF > 0.2 and little or no missing data were used to estimate the relative kinship in the set of 143 lines. As shown in Figure 5, the average relative kinship between any two lines was 0.0332, or ~57% of the pairwise kinship estimates were close to 0, and 21% of the kinship estimates ranged from 0 to 0.05. The remaining estimates ranged from 0.05 to 1, with a continuously decreasing number of pairs falling in higher estimated categories. These results indicated that most lines in the panel have no or very weak kinship, which might be attributed to the broad ranging collection of genotypes and the exclusion of similar genotypes before analysis.

### Association mapping and candidate gene prediction

**Figure 6:**
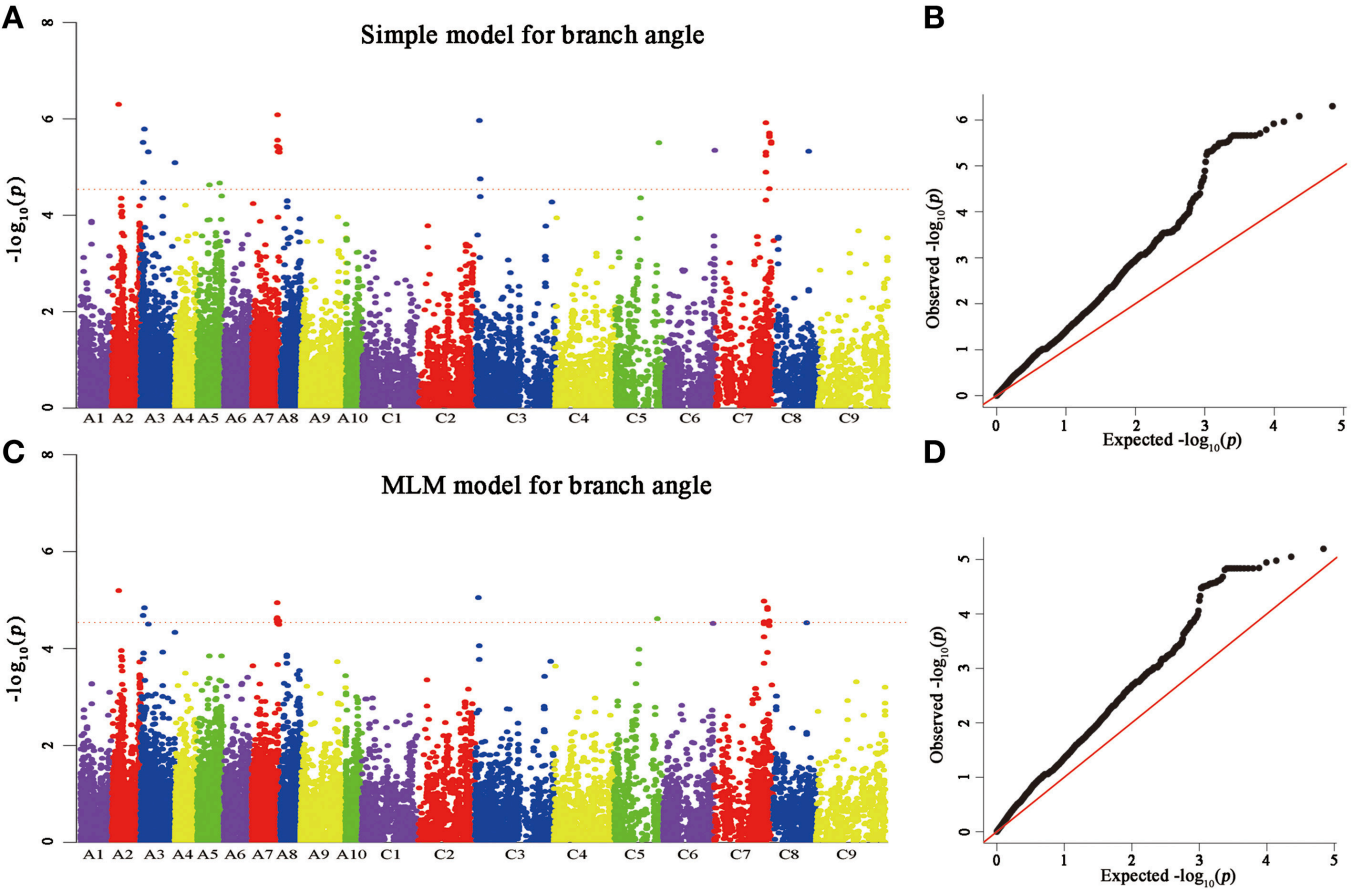
**Genome-wide association analysis of branch angle. (A)** Manhattan plots of the simple model for BA. **(B)** A quantile-quantile (QQ) plot for a simple branch model **(C)** Manhattan plots of MLM for BA, as in **(A)**. **(D)** A quantile-quantile (QQ) plot of MLM for BA. Each dot represents a SNP. The horizontal dashed red line indicates the Bonferroni-corrected significance threshold at -log_10_(*P*) = 4.5.

A total of 25 and 60 associations (*P* < 2.90E^−5^) were detected for branch angle by using BLUP across three environments and an individual environment (Figure 6, Table 3; Supplementary Figure 2). To select the major QTL among all the significant SNPs, these SNPs were clumped by using an LD block as a criterion (Gabriel et al., 2002), and the peak SNP within each LD block was retained. After the clumping of SNPs, six QTL for branch angle were distinguished with the BLUP values of a branch angle across three environments and the peak SNPs are listed in Table 3. The six peak regions were 6.1, 3.5, 23.2, 3.0, 39.5, and 48.7 Mb of A2, A3, A7, C3, C5, and C7, respectively, in the “pseudomolecules” of *B. napus*, and the cumulative phenotypic variance explained by all significant SNPs was 92.42%, which contributed to 16.60–18.95% of the phenotypic variance based on the *R*^2^-values. In Yangluo-2013, nine peak SNPs were detected in Q+K models with an FDR of 0.021, and the eight peak regions were 10.1, 22.2, 18.0, 0.1, 42.8, 48.7, and 2.9 Mb for A2, A5, A8, A10, C2, C6, C7, and C8, respectively. In Yangluo-2014, two peak SNPs were detected with FDRs of 0.013 and 0.027, and the two peak regions were 48.7 and 23.2 Mb of A7 and C7, respectively. In Lu'an-2013, only one significantly peak SNP in A3 was detected in 31.3 Mb region with an FDR of 0.263.

**Table 3:** **A summary of significant (***P*** < 2.90E^**−5**^) SNP-trait associations associated with branch angle**.

**Environment**	**Peak SNP name**	**Chr^a^**	**Position^b^ (kb)**	***P***	**MAF^C^**	***R*^2^ (%)**	**Candidate gene**	**Marker distance from candidate gene (kb)^d^**
BA-BLUPS	Bn-A02-p7183200	A2	6140	6.38E-06	(A)0.084	18.93		
	Bn-A03-p3571859	A3	3571	1.46E-05	(G)0.310	17.53	*BnaA.Lazy.A3*	488 (Upstream)
	Bn-A07-p19977445	A7	23,182	1.14E-05	(G)0.196	18.58	*BnaA.SPL14.A7*	521 (Downstream)
	Bn-scaff_18936_1-p210783	C3	2962	8.92E-06	(A)0.286	18.41	*BnaC.Lazy.C3*	243 (Downstream)
	Bn-scaff_21369_1-p378189	C5	39,469	2.43E-05	(G)0.304	16.60		
	Bn-scaff_16110_1-p1940585	C7	48,687	1.43E-05	(C)0.141	17.56	Auxin-responsive GH3 family protein	4 (Upstream)
BA-13YL	Bn-A02-p11153317	A2	10,098	1.43E-05	(A)0.268	17.57	*BnaA.SPL14.A2*	898 (Downstream)
	Bn-A05-p22938228	A5	22,197	2.61E-05	(A)0.185	16.54		
	Bn-A08-p20838949	A8	18,001	2.75E-06	(C)0.077	20.51		
	Bn-A10-p4727374	A10	137	1.03E-05	(G)0.152	18.14		
	Bn-scaff_15712_2-p770819	C2	42,843	2.10E-05	(A)0.325	17.00		
	Bn-scaff_17799_1-p1178394	C6	45,148	2.61E-05	(G)0.120	18.71	*BnaC.SPL14.C6*	11 (Downstream)
	Bn-scaff_16110_1-p1939090	C7	48,689	2.72E-05	(G)0.129	16.47	Auxin-responsive GH3 family protein	6 (Upstream)
	Bn-scaff_21003_1-p435731	C8	2901	1.90E-05	(A)0.259	17.08		
BA-14YL	Bn-scaff_16110_1-p1940470	C7	48,687	5.43E-06	(A)0.140	19.84	Auxin-responsive GH3 family protein	4 (Upstream)
	Bn-A07-p19977445	A7	23,182	2.15E-05	(G)0.196	18.31		
BA-13LA	Bn-A03-p31327366	A3	31,323	2.90E-05	(A)0.129	16.64		

a Chromosome;

b The physical position of SNP is inferred from BLAST hits of the chromosome pseudomolecules in B. napus;

c Minor allele frequency;

d *The marker distance and its upstream or downstream from candidate gene*.

Notably, the *Lazy* orthologs were searched in the “pseudomolecules” of *B. napus* and two orthologs were found at 2.6 Mb in A3 and 3.2 Mb in C3, which were 488 kb away from the peak SNP Bn-A03-p3571859 and 243 kb away from the peak SNP Bn-A03-p3571859. In addition, the *Squamosa Promoter Binding Protein-like* 14 (*SPL14*) orthologs were searched in the “pseudomolecules” of *B. napus* and one ortholog was found at 24.3 Mb of A7, which was 521 kb away from the peak SNP Bn-A07-p19977445. Aside from the *Lazy* and *SPL14* genes, an auxin-responsive *GRETCHEN HAGEN 3* (*GH3*) ortholog “pseudomolecules” of *B. napus* in 48.7 Mb of C7, which was very close (only 4 kb) to the peak SNP Bn-scaff-16110-1-p1940585.

## Discussion

In this study, a panel comprising 143 *B. napus* germplasm lines was adopted for association mapping study. Significant natural phenotypic variation in branch angle was observed in rapeseed germplasms. In general, to unravel the genetic basis of trait variation, a number of traditional genome-wide phenotype-to-genotype approaches have been employed by LD association mapping. An association mapping study employs the large number of recombination events that occur throughout the entire breeding selection history of the mapping population, thereby allowing fine-scale QTL mapping (Nordborg and Tavaré, 2002). Although the sample size is not sufficiently large in our association panel, the phenotypic variation in branch angle is very large. The heritability of this trait is relatively high, and it is related to significant genome loci with great effects. However, there must be existence of some unknown genes regulating branch angle in rapeseed comparing to model plant *Arabidopsis* and rice (Teichmann and Muhr, 2015), which still could not make of it. The novelty SNP clusters of QTL from our preliminary exploration are starting to decompose this aspect. Based on the MLM model, a total of 60 SNP associations (*P* < 2.90E^−5^) were detected for branch angle in three environments, and 25 significant SNP loci were further verified using BLUP model. Through the analysis of GWA, the markers detected in the environmental BLUPs value correlated significantly (*P* < 0.001) with branch angle, with phenotypic value effects between 16.60 and 18.93%. Exploring these associated markers provides a genetic basis to analyze branch angle variation in rapeseed.

The discovery of many false-positive QTL is due to the population structure (Zhao et al., 2007). To resolve this problem, several models have been developed including the Q+K model and the PCA model. Although the Q+K model have been demonstrated the most powerful method for identifying associations by many studies (Yu et al., 2005; Stich and Melchinger, 2009), we also compared different models and obtained the same conclusion that the MLM model (Q+K) was most suitable for our population. In our study, all *B. napus* accession lines can be largely divided into two sub-populations in STRUCTURE and compared with an association analysis from a previous study (Harper et al., 2012), and the population structure classification leads to the same results. Hence, the reasonable results for a population structure-associated analysis provide a foundation and guarantee. For the association analysis, we used a mixed model approach that avoided a confounding effect in the population structure and population relations. We acquired the branch angle BLUP values in three independent environments to eliminate the influence of environment.

To account for the potential number of false positives, we implemented stringent quality control on the included polymorphisms, with conservative non-parametric testing and an adjusted statistical significance threshold. Our approach relied on natural variations in rapeseed, leading to a set of strong candidate genes by comparative genome method. A high degree of co-linearity and congruence in the *A. thaliana* and *Brassica* genomes has been recognized (Parkin et al., 2005; Ziolkowski et al., 2006) and a single locus in the *Arabidopsis* genome is generally represented by three distinct loci in diploid *Brassica* species. Several genes that play key roles in regulating branch development in *Arabidopsis* have been identified. By applying this method to the recently grown tetraploid crop *B. napus*, we identified genomic regions that underlie four QTL for branch angle. The detected regions contained two *Lazy* (AT5G14090) orthologous, *SPL14* (AT1G20980) and Auxin-responsive *GH3* family protein (AT5G51470) genes, which were all relevant to angle formation in *Arabidopsis thaliana*.

Rapeseed is an excellent model for association analyses because of the extensive architectural variation across its native range and its diverse germplasm collections through artificial selection. During rapeseed domestication and breeding process, breeders often focus on important characteristics such as oil content, biotic stress resistance, and quality for directional selection for greater oil yield and better quality, which were directly caused by an artificial selection gene locus mutation at a lower frequency. For example, in terms of oil and glucosinolate contents, and because of the artificial selection on *Fatty Acid Elongation 1* (*FAE1*) and *High Aliphatic Glucosinolate 1* (*HAG1*) gene locus, gene locus mutation occurs at a lower frequency in double-low rapeseed populations (Li et al., 2014). However, the optimation of rapeseed architecture (branch angle and plant height) to improve yield is more urgent nowadays. Association-mapping approach has advantage for distinguishing the most favorable alleles within a diverse genetic background, which provides the necessary genotypic information to facilitate the design of efficient rapeseed introgression and selection schemes throughout the world. This study indicated that the SNP variation frequency control of rapeseed branch angle habits is slightly higher than the results. This finding may be related to the domestication and genetic improvement of characteristics that are not particularly selected by the breeding program.

The association of branch angle with increased plant productivity in rapeseed may be beneficial to lodging resistance and high-density field cultivation. This genetic analysis of branch angle and plant morphological characteristics was only based on one natural population. Therefore, the observed inter-trait and trait-marker associations must be confirmed in other rapeseed populations. Further evaluations of branch angle and plant morphology should be conducted under contrasting cropping density, and total seed yield measurements should be taken because the seed harvest index in addition to the plant growth and productivity are strongly affected by the cropping density.

## Conclusion

In this study, GWA mapping with corrections for the population structure were used to identify a number of novel loci and refine the map locations of known loci related to branch angle in rapeseed. This information not only demonstrates that GWAS mapping can be used as a powerful tool for dissecting plant architecture mechanisms in rapeseed but also provides valuable markers for breeding rapeseed cultivars with the ideotype. In addition, the candidate genes nearby these SNP loci represent promising targets for efforts to further identify causal variants and to clarify how the implicated genes affect branch angle in rapeseed.

## Author contributions

JL and QH conceived and designed the study. JL, WW, and DL conducted the experiments; DM and HW coordinated genotyping with SNP markers; LF and YL provided rapeseed lines; JL and WW analyzed and interpreted data, and prepared the manuscript; QH supervised the whole study; all authors reviewed and edited the manuscript.

### Conflict of interest statement

The authors declare that the research was conducted in the absence of any commercial or financial relationships that could be construed as a potential conflict of interest.
