# Supplementary material for: Characterizing Variation of Branch Angle and Genome-Wide Association Mapping in Rapeseed (Brassica napus L.)
Source: Front Plant Sci. 2016 Feb 4;7:21. doi: 10.3389/fpls.2016.00021 (PMC4740498; doi:10.3389/fpls.2016.00021)
Supplement: Supplementary file 3 [file DataSheet1.DOC]

**
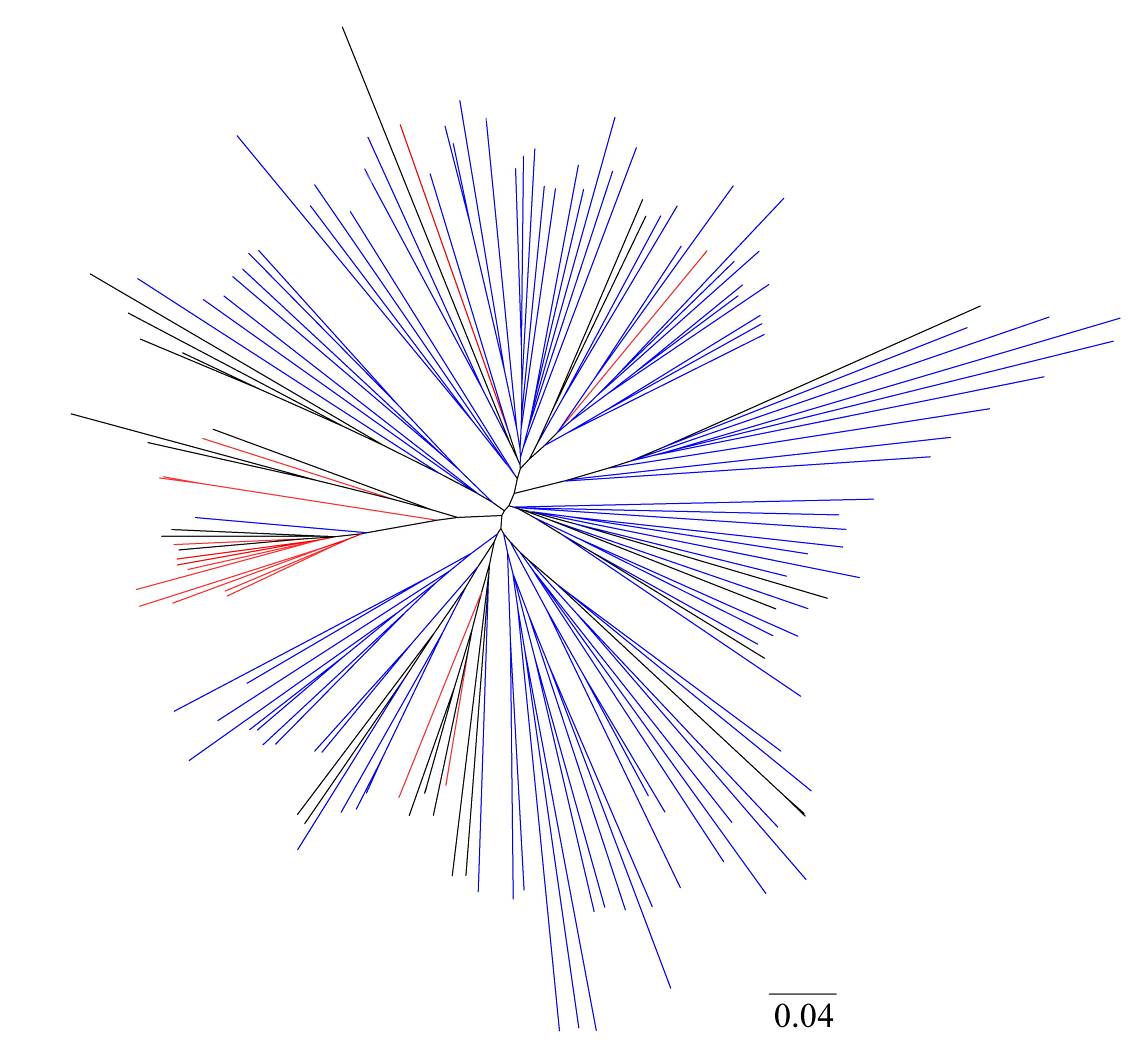
Supplementary Figure 1.** A neighbor-joining phylogenetic tree based on Nei’s genetic distance. Red states for Group 1, blue for Group 2, and black for the mixed group.
