# Supplementary material for: Characterizing Variation of Branch Angle and Genome-Wide Association Mapping in Rapeseed (Brassica napus L.)
Source: Front Plant Sci. 2016 Feb 4;7:21. doi: 10.3389/fpls.2016.00021 (PMC4740498; doi:10.3389/fpls.2016.00021)
Supplement: Supplementary file 4 [file DataSheet2.DOC]

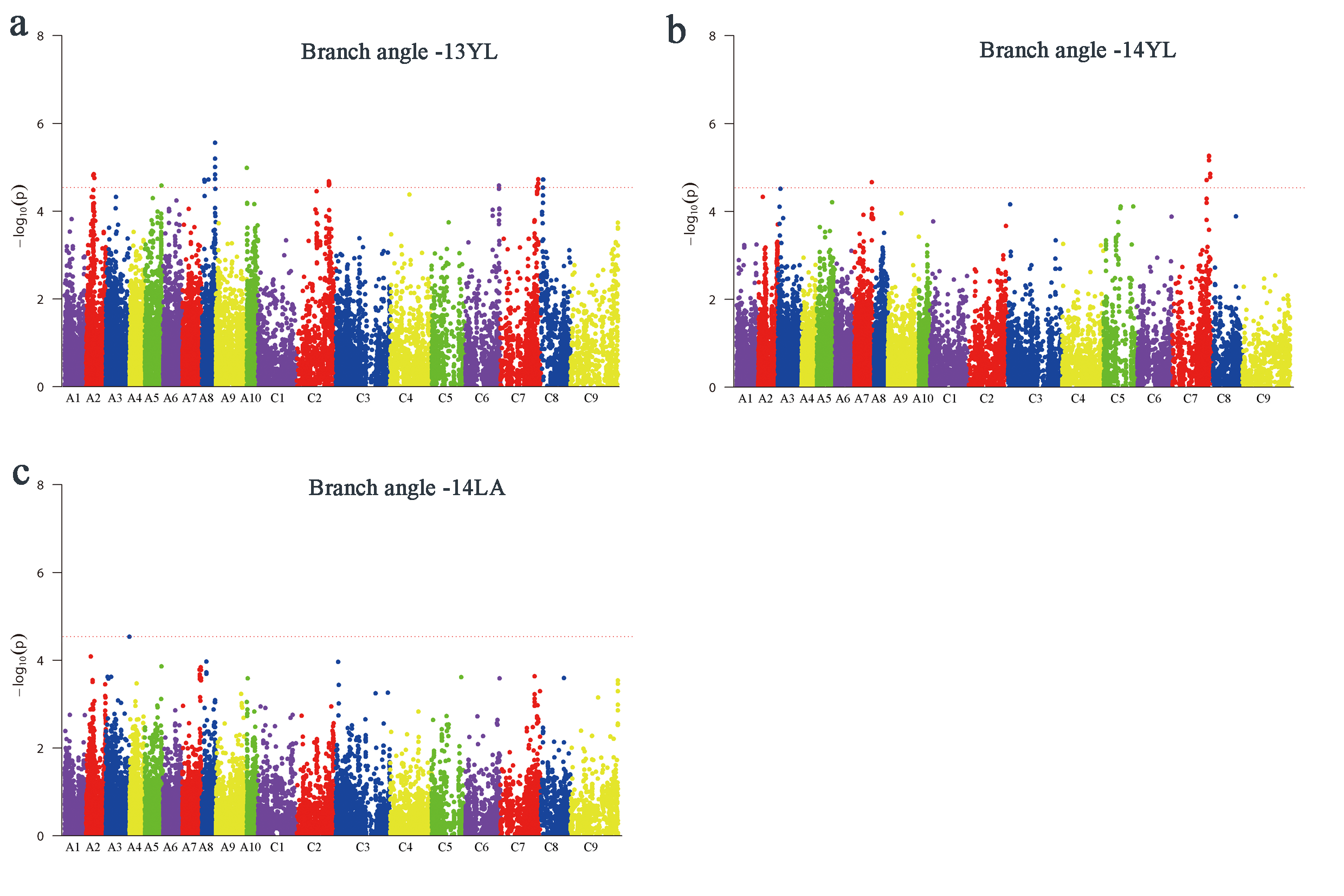
**Supplementary Figure 2.** Genome-wide association analysis of branch angle in three individual environments. (a) (b) (c) Manhattan plots of MLM for BA in 13YL, 14YL and 14LA, respectively.
